# Diagnostic Accuracy of Serum Procalcitonin for Spontaneous Bacterial Peritonitis Due to End-stage Liver Disease: A Meta-analysis

**DOI:** 10.1097/MD.0000000000002077

**Published:** 2015-12-11

**Authors:** Yongtao Yang, Lianyong Li, Changmin Qu, Bolun Zeng, Shuwen Liang, Zhiwen Luo, Xiaoying Wang, Changqing Zhong

**Affiliations:** From the Department of Gastroenterology, The 306th Hospital of PLA, Chaoyang District, Beijing, China.

## Abstract

Early diagnosis and prompt treatment of spontaneous bacterial peritonitis (SBP) due to end-stage liver disease is vital to shorten hospital stays and reduce mortality. Many studies have explored the potential usefulness of serum procalcitonin (PCT) in predicting SBP. The aim of this study is to evaluate the overall diagnostic accuracy of PCT levels for identifying SBP due to end-stage liver disease.

After performing a systematic search of the Medline, Embase, and Cochrane databases for studies that evaluated the diagnostic role of PCT for SBP, sensitivity, specificity, and other measures of accuracy of PCT concentrations in serum for SBP diagnosis were pooled using random-effects models. A summary receiver operating characteristic curve was used to summarize overall test performance.

Seven publications met the inclusion criteria covering 742 episodes of suspected SBP along with 339 confirmed cases. The summary estimates for serum PCT in the diagnosis of SBP attributable to end-stage liver disease were: sensitivity 0.82 (95% CI 0.79–0.87), specificity 0.86 (95% CI 0.82–0.89), positive likelihood ratio 4.94 (95% CI 2.28–10.70), negative likelihood ratio 0.22 (95% CI 0.10–0.52), and diagnostic OR 22.55 (95% CI 7.01–108.30). The area under the curve was 0.92. There was evidence of significant heterogeneity but no evidence of publication bias.

Serum PCT is a relatively sensitive and specific test for the identification of SBP. However, due to the limited high-quality studies available, medical decisions should be carefully made in the context of both PCT test results and other clinical findings.

## INTRODUCTION

Bacterial infections account for significant morbidity and mortality in end-stage liver disease (ESLD) patients. Spontaneous bacterial peritonitis (SBP) is the most frequent and life-threatening infection in these patients.^1^ Cirrhotic patients with SBP frequently develop a rapidly progressive impairment in systemic hemodynamics, leading to severe renal and hepatic failure, aggravation of portal hypertension, encephalopathy, and death.^2^ When first described, its mortality exceeded 90%, but it has been reduced to ∼20% with early diagnosis and treatment.^3^ Early diagnosis of SBP is essential in hospitalized patients with liver disease; however, this presents a challenge for clinicians because of the frequent lack of symptoms in the early stages of SBP.^4^ At present, most guidelines recommend that a diagnostic paracentesis should be performed in all patients with ascites admitted to hospital regardless of whether or not there is clinical suspicion. Diagnosis is established by an ascites polymorphonuclear cells (PMN) count >250 cells/mm.^5^ Although paracentesis is usually a safe procedure, complications may occur from time to time. It has been reported that abdominal wall hematomas in ∼1% of patients, and even more severe complications such as hemoperitoneum or bowel penetration by the needle occurred in 1 case per 1000 paracenteses.^6^ In rare cases, pathogenic bacteria such as staphylococci can be introduced along with the needle to the ascites. It is therefore clear that using blood for routine examinations is more convenient and safer than ascitic fluid, and the measurement of serum biomarkers has consequently received much attention recently for the early diagnosis of SBP.

Procalcitonin (PCT) is a precursor of calcitonin, normally secreted by C cells of the thyroid in healthy individuals. In the absence of infection, the extra-thyroidal expression of the PCT gene in the liver, lung, kidney, adrenal tissue, monocytes, granulocytes, testis, prostate gland, and small intestine is suppressed.^7^ Procalcitonin in the blood of healthy individuals is below the limit of detection of clinical assays, but microbial infection, especially of bacterial origin, induces a ubiquitous increase in PCT gene expression, resulting in the constitutive release of PCT from parenchymal tissues throughout the body. In bacterial infections, serum PCT levels start to rise 4 h after the onset of systemic infection and peak between 8 and 24 h.^8^ The half-life of PCT in serum is 20 to 24 h, which makes it suitable for early detection and daily monitoring.^7^

Several studies have investigated the diagnostic value of PCT in patients with SBP due to liver disease.^9–11^ Results on the accuracy of PCT in the early detection of bacterial infection in liver cirrhosis, especially SBP, are controversial. In 2013, one study performed meta-analysis and reported moderate to high accuracy for PCT as a diagnostic aid for SBP.^12^ Since then, other studies of PCT have increased our understanding further.^13–16^ Here, we performed meta-analysis to explore the ability of serum PCT to differentiate infected from uninfected ascites in ESLD patients.

## MATERIALS AND METHODS

### Search Strategy and Study Selection

A comprehensive search of the Medline (PubMed was used as the search engine), Embase and the Cochrane databases was performed to identify suitable articles up until 30 January 2015. The search was not restricted to any particular language. The related-articles function in PubMed was used to identify relevant articles. The bibliographies of retrieved articles were also searched to identify further relevant studies manually. The Medline search used a combination of terms “procalcitonin,” “PCT,” “peritonitis,” “ascites,” “liver disease,” “cirrhosis,” and “severe hepatitis.” Similar strategies were used for searching the other databases. For a study to be included in the meta-analysis it had to be with sufficient information for constructing a 2 × 2 contingency table, meaning that both false and true positives and negatives were provided for the diagnosis of SBP. Data extraction and quality control were performed by 2 reviewers (YTY and LYL) for each selected study. Disagreements were resolved by consensus. The authors were contacted and asked to supply further details when data on specified outcomes or individual patients were absent. Ethics approval was not needed for this study because it was based on the previous published data.

### Data Extraction and Quality Assessment

Data extracted from the reports included authors, publication year, age, sex, etiology, severity of liver disease, cut-off value, methodological quality, and diagnosis of SBP. Prospective and retrospective study designs were retrieved. To evaluate the study quality and potential for bias, quality assessment was conducted using the Quality Assessment of Diagnostic Accuracy Studies-2 (QUADAS-2) tool. The QUADAS-2 tool comprises 4 domains, which are patient selection, index test, reference standard, and flow and timing. The risk of bias was analyzed and rated as low risk, high risk, or unclear risk for each domain. The first 3 domains are also assessed in terms of concerns regarding applicability and also rated as low risk, high risk, or unclear risk. The quality assessment present with an overall evaluation of the quality of the included studies, which is helpful to explore potential sources of heterogeneity.^17^

### Statistical Analyses

Standard methods recommended for meta-analyses of diagnostic test evaluations were used.^18^ We performed data analysis using Meta-DiSc software (version 1.4). The measures of diagnostic accuracy including sensitivity, specificity, positive likelihood ratio (PLR), negative likelihood ratio (NLR), and diagnostic OR (DOR) were calculated for each study.

Summary receiver operating characteristic (sROC) analysis based on Moses and colleagues’ method was used to reflect the discriminating ability of the diagnostic test.^19–20^ The sROC curve is a plot of the true positive rate (sensitivity) as a function of the false positive rate (1-specificity).^18^ An sROC curve is constructed based on a linear model to fit these points. To construct the linear model, 1 has to transform 2 parameters, D and S, using the true positive rate (TPR) and false positive rates (FPR), of which D = ln(TPR/[1−TPR]) – ln(FPR/[1−FPR]) and S = ln(TPR/[1−TPR]) + ln(FPR/[1−FPR]). The constructed linear regression equation to fit these points is as follows: D = β×S+α, in which α is used as the y-intercept, whereas β is the regression coefficient of dependent variable D to independent variable S.^18^

To explore the heterogeneity across the studies, the chi square and Fisher exact tests were used. A random effects model was used to compute the pooled sensitivity, specificity, and other estimates across studies. Sensitivity analyses were performed on important diagnostic measures if heterogeneity existed. Subgroup analysis was performed for prospective versus retrospective studies to examine the effect of study types on the results. Furthermore, to assess the role of disease types for ESLD on the results, a subgroup analysis on studies of patients with cirrhosis versus studies of patients with severe hepatitis was performed. Levels of significance were measured at *P* < 0.01. The potential publication bias for meta-analyses was evaluated using funnel plots and the Egger test.^21^ STATA software (version 11.0, College Station, TX) were used to perform the analyses.

## RESULTS

**FIGURE 1:**
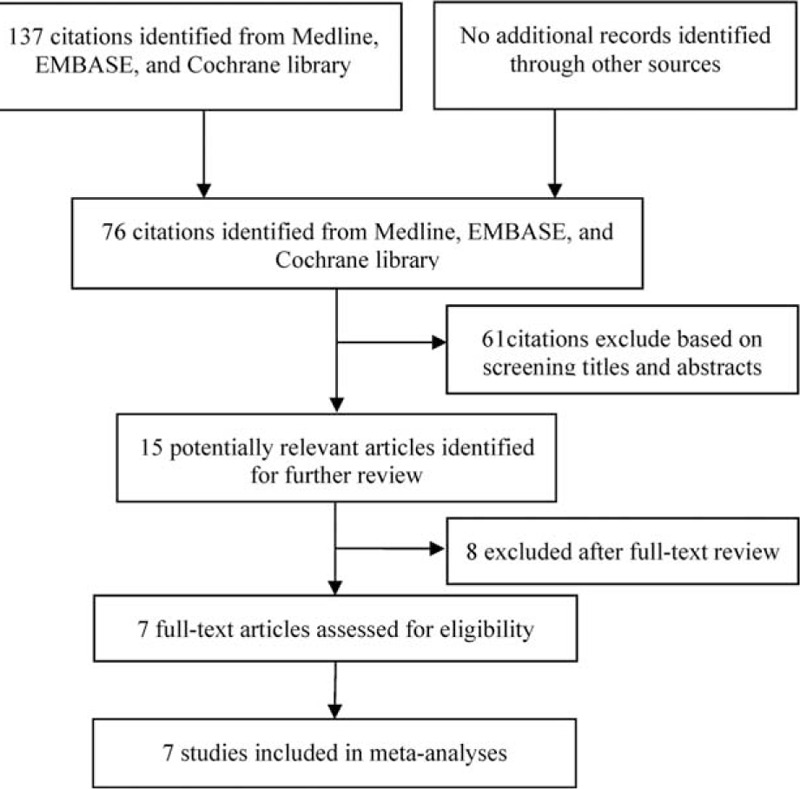
Flowchart showing the process for selecting eligible studies in this meta-analysis.

In total, 76 studies were identified using the described search strategies. After the first round screening of title and abstracts, 61 non-relevant studies, case reports, and reviews were excluded. Fifteen potentially relevant studies were retrieved for full-text evaluation, of which 8 further studies were excluded because 5 did not study or report outcomes of interest, 2 had insufficient data to reconstruct 2 × 2 tables, and 1 studied on secondary petitonitis. Finally, 7 publications determining PCT concentrations in serum in patients with SBP were considered to be eligible for inclusion in the analysis ^9–11,13–16^ (Fig. 1). These studies included 742 episodes of suspected infection, with 339 (45.6%) confirmed episodes. Two studies reported data using the standard PCT cut-off value (0.5 ng/mL).^10,14^ Five studies used the optimized threshold with best sensitivity and/or specificity.^9,11,13,15–16^ The number of subjects in each study ranged from 20 to 362. The age, sex, etiology, and severity of liver disease and others characteristics in the included studies were recorded and summarized in Table 1. All articles included in our analysis had well-described inclusion and exclusion criteria. Spontaneous bacterial peritonitis was defined as an ascites sample with a PMN cell population of >250 cell/mm^3^ with or without culture positivity, or compatible clinical symptoms. Other sites of bacterial infection, including pneumonia or meningitis, or positive bacterial culture in blood, urine, or sputum were excluded.

**TABLE 1:**
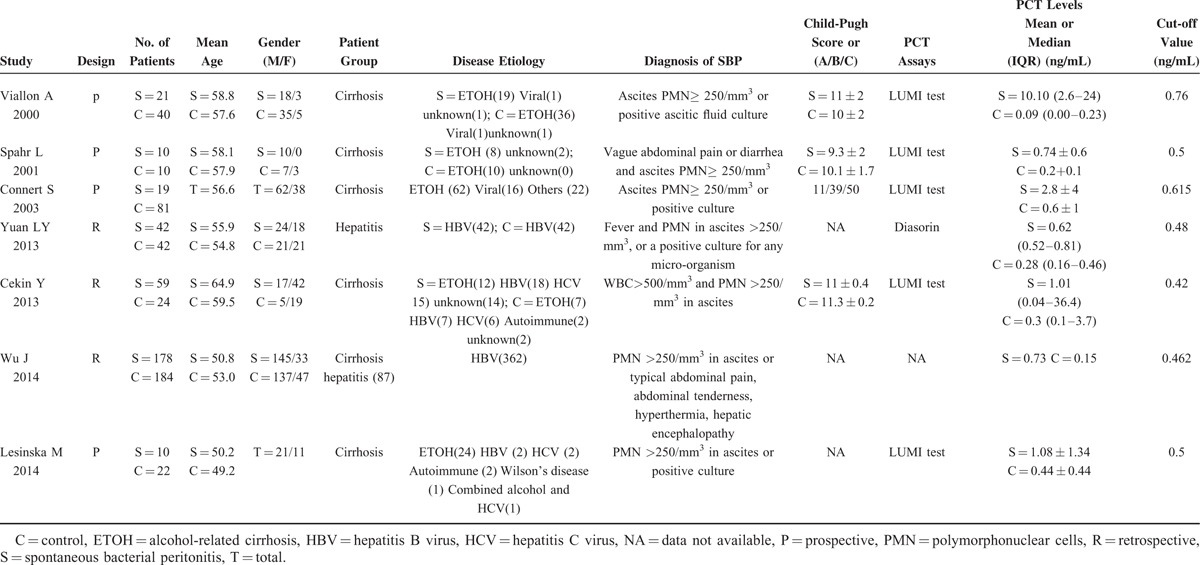
Description of Individual Studies Included in the Meta-Analysis

### Methodological Quality of Included Studies

**FIGURE 2:**
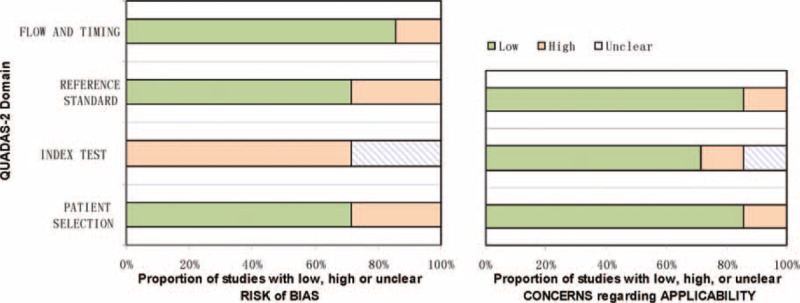
Methodological evaluation of the included studies according to QUADAS-2. Graphical display of the QUADAS-2 results showing the proportion of studies with high, low, or unclear risk levels of bias.QUADAS-2 = Quality Assessment of Diagnostic Accuracy Studies-2.

Evaluation of the risk of bias and concerns regarding applicability of the selected studies were performed according to QUADAS-2 criteria. Concerning the domain of patient selection bias, 2 retrospective studies did not explicitly report that patients were consecutives.^13,16^ As to the domain of the index test, 5 studies selected a test threshold with optimized sensitivity and/or specificity based on the ROC curve, which may lead to overestimation of test performance.^9,11,13,15–16^ Regarding applicability concerns for the index test, 6 studies measured PCT levels using the widely commercially available immuno-luminometric assay (LUMItest PCT; distributed by BRAHMS Diagnostica, Berlin, Germany), whereas one study used automated immunoanalysis of different sources (Diasorin, Saluggia, Italy).^15^ Regarding the reference standard, one study excluded bacterascites (white blood cell count < 500/mm^3^ and PMN <250/mm^3^ in ascites with a positive bacterial culture) as SBP, whereas one study diagnosed SBP based on clinical symptoms without paracentesis of patients.^13,16^ Regarding the domain of flow and timing, one study did not report the interval between the index test and the final diagnosis.^13^ The QUADAS-2 results of quality assessment for the 7 studies were presented graphically showing the proportion of studies with low, high, or unclear risks of bias (Fig. 2).

### Diagnostic Accuracy

**FIGURE 3:**
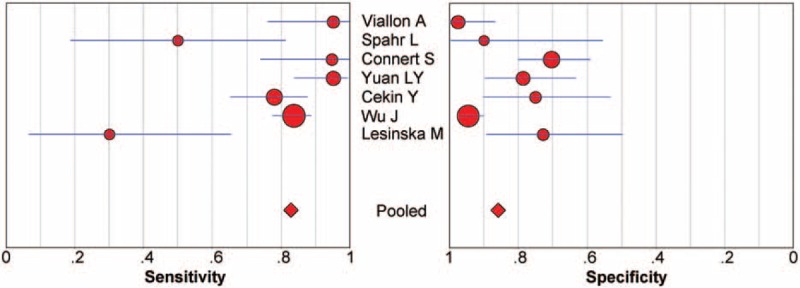
Forest plot of estimates of sensitivity and specificity for procalcitonin assays in the diagnosis of SBP. The point estimates of sensitivity and specificity from each study are shown as solid circles. Error bars indicate 95% CIs. CI = confidence interval, SBP = spontaneous bacterial peritonitis.

Figure 3 shows the forest plot of sensitivity and specificity for the 7 studies for the PCT assays used to diagnose SBP due to ESLD. The sensitivity varied greatly, ranging from 0.30 to 0.95 (pooled 0.82, 95% confidence interval [CI] 0.79–0.87), whereas specificity ranged from 0.70 to 0.98 (pooled 0.86, 95% CI 0.82–0.89). It was of note that the pooled PLR was 4.94 (95% CI 2.28–10.70), the NLR was 0.22 (95% CI 0.10–0.52), and the DOR was 22.55 (95% CI 7.01–108.30) (Table 2). Chi-square values for sensitivity, specificity, PLR, NLR, and DOR were 31.76, 40.09, 43.35, 62.66, and 33.76, respectively. *P* values of sensitivity, specificity, PLR, NLR, and DOR were all <0.01, indicating significant heterogeneity existed across studies concerning sensitivity, specificity, PLR, NLR, and DOR.

**TABLE 2:**
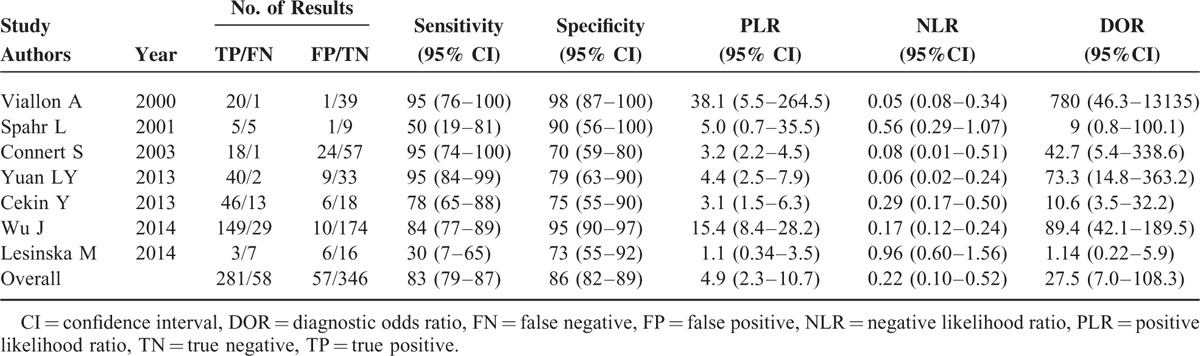
Results Derived from the 2 × 2 Tables of Individual Studies Using Serum Procalcitonin as a Marker for SBP

**FIGURE 4:**
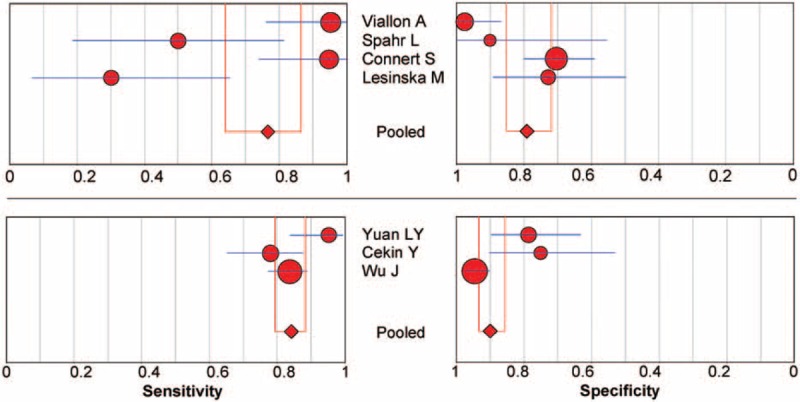
Subgroup analysis of prospective versus retrospective studies of procalcitonin in the diagnosis of SBP. The point estimates of sensitivity and specificity from each study are shown as solid circles. Error bars indicate 95% CIs. CI = confidence interval, SBP = spontaneous bacterial peritonitis.

Sensitivity analysis based on DOR was performed by excluding studies one by one to investigate the heterogeneity. However, the analysis results showed that no individual studies significantly affected the pooled DOR (data not shown). Subgroup analysis of pooled sensitivity and specificity for the 4 prospective studies gave values of 0.77 (95% CI 0.64–0.87, *P* < 0.001) and 0.79 (95% CI 0.72–0.85, *P* = 0.0008), whereas the pooled sensitivity and specificity values for the 3 retrospective studies were 0.84 (95% CI 0.79–0.88, *P* = 0.0358) and 0.90 (95% CI 0.86–0.93, *P* = 0.0008) (Fig. 4), indicating a significant degree of heterogeneity for both the prospective and the retrospective studies.

**FIGURE 5:**
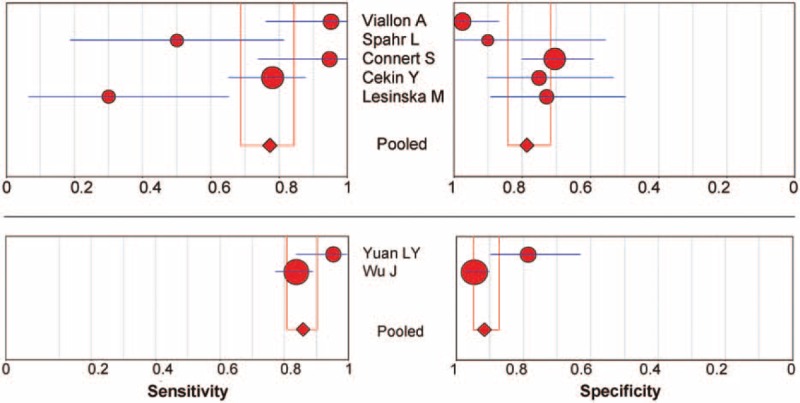
Subgroup analysis of underlying diseases on the effect of procalcitonin in the diagnosis of SBP. The point estimates of sensitivity and specificity from each study are shown as solid circles. Error bars indicate 95% CIs. CI = confidence interval, SBP = spontaneous bacterial peritonitis.

There were 5 studies in patients with cirrhosis only, 1 in patients with both cirrhosis and severe hepatitis, and 1 in patients with severe hepatitis B only. Subgroup analysis of the pooled sensitivity and specificity values for the 5 studies in patients with cirrhosis were 0.77 (95% CI 0.69–0.85, *P* < 0.001) and 0.79 (95% CI 0.72–0.84, *P* = 0.002), whereas the pooled sensitivity and specificity values for the 2 studies containing patients with severe hepatitis were 0.86 (95% CI 0.81–0.90, *P* = 0.032) and 0.92 (95% CI 0.87–0.95, *P* = 0.003) (Fig. 5), indicating significant heterogeneity.

**FIGURE 6:**
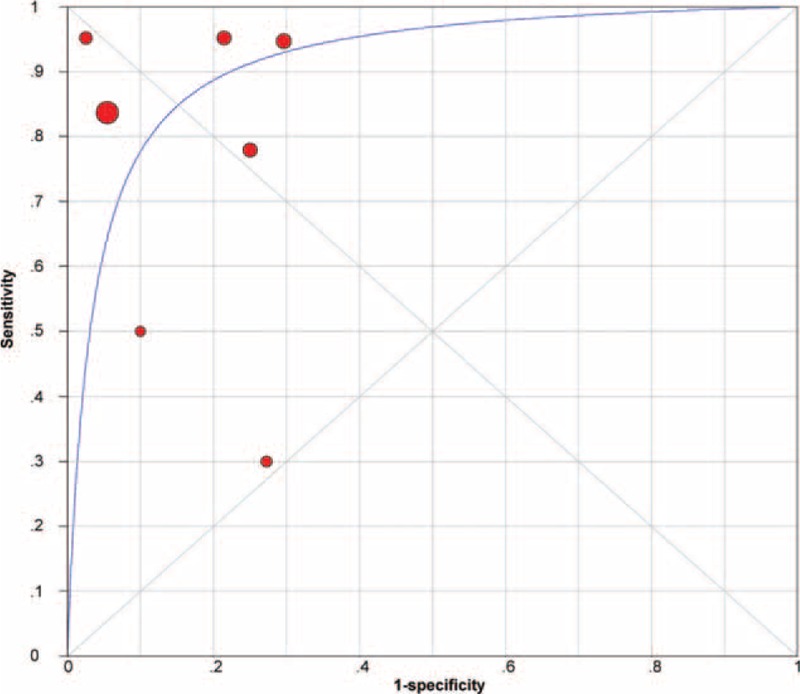
Summary receiver operating characteristic curves summarize the overall diagnostic accuracy for procalcitonin assays. Each solid circle represents each study in the meta-analysis. Symbol size for each study is proportional to the study size.

A graph of the SROC curve for serum PCT determination showing TPRs versus FPRs from individual studies is shown in Figure 6. The difference in sample size among the studies was taken into account by weighing each study by the reciprocal of the variance of difference and performing weighted regression. The index Q and area under curve (AUC) values from the SROC curves were calculated; Q value corresponds to the upper most point on the SROC curve in which sensitivity (or true positivity) equals specificity.^22^ A higher Q value indicates higher accuracy. In the present study, the Q value was 0.85 (SEM 0.049), indicating the maximum joint sensitivity and specificity of PCT for SBP was 0.85. The AUC represents an analytical summary of test performance and displays the trade-off between specificity and sensitivity.^18^ An AUC of >0.9 indicates high diagnostic accuracy. In the present meta-analysis, the AUC was 0.92, indicating that PCT assays had a moderate to high degree of overall accuracy.

### Publication Bias

**FIGURE 7:**
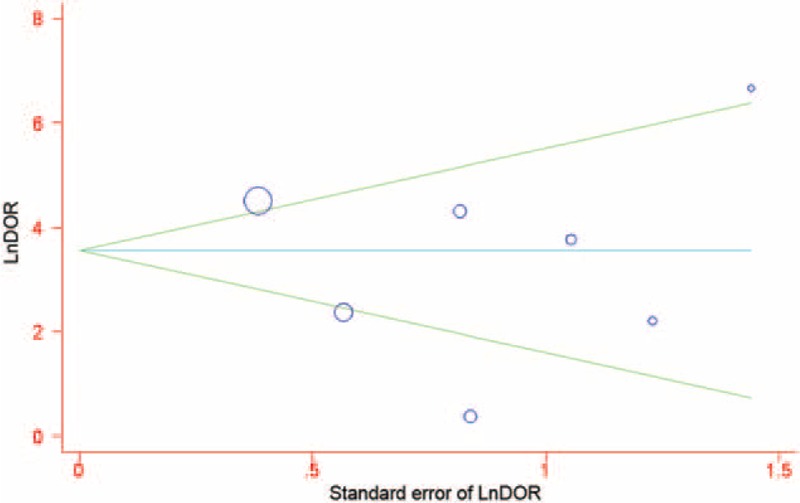
Funnel graph for the evaluation of potential publication bias in procalcitonin assays. Symbol size for each study is proportional to the study size. The line in the center indicates the summary DOR. The Egger test for publication bias was not significant. DOR = diagnostic odds ratio.

The potential publication bias of the meta-analysis was analyzed using Begg's funnel plot and Egger's test. Begg's test showed a *P* value of 0.88. Egger's test showed a value of −1.11 (95% CI −6.93 to 4.70, *P* = 0.643) on a per-patient analysis (Fig. 7). Both of which indicated that there was no potential publication bias.

## DISCUSSION

Spontaneous bacterial peritonitis has been acknowledged as a potential trigger factor in many complications of cirrhosis, including variceal bleeding, hepatic encephalopathy, renal failure, and impairment in clotting factors, worsening hepatic status and leading to death in patients with cirrhosis.^23^ The usual absence of typical clinical characteristics in this patient population makes identification of SBP difficult.^24^ An ascitic PMN count >250 cells/mm^3^ is considered diagnostic of SBP and triggers immediate initiation an empirical antibiotic treatment.^3^ Spontaneous bacterial peritonitis caused by Gram-positive cocci is associated with a PMN count below the threshold of 250 cells/mm^3^, because the stimulatory capacity for PMN migration depends on the type of bacteria. The suitability of a range of biomarkers, such as serum C-reactive protein, PCT, lipopolysaccharide-binding protein, ascites leucocyte esterase activity, lactoferrin or bacterial DNA, to be used in rapid screening tests has been explored in many studies.^23,25–28^ At present, the diagnostic accuracy of these methods vary greatly between studies and further investigations are needed to evaluate their discriminative capability.

There is considerable evidence indicating that high PCT levels are related to infection in cirrhosis.^9,29^ However, the accuracy of using PCT levels for the early detection of infection, especially for SBP, in ESLD patients is controversial.^30–31^ In some studies, serum PCT levels were reported to diagnose SBP with satisfactory sensitivity and specificity, whereas in others the diagnostic values were doubtful. A previous systematic review included 3 studies on cirrhosis patients published between April 2000 and February 2003 investigating the diagnostic performance of PCT as a marker of SBP. They reported good diagnostic performance, with an AUC of 0.95 (95% CI 0.82–0.99) and a DOR of 59.9 (95% CI 5.78–619.8).^12^ They concluded that both positive and negative likelihood ratios showed high discriminative ability and could be used as a valuable tool for rule-in and rule-out SBP diagnosis.^12^ However, due to the limitations of the existing literature, they could not draw a firm conclusion on the diagnostic performance of PCT testing.

We updated these findings by performing meta-analysis on more recent related studies. Our analysis included 7 studies with large sample sizes and balanced baseline characteristics. We report a mean sensitivity for PCT of 0.82, a mean specificity of 0.86, a Q value from the SROC of 0.85, and an AUC of 0.92, indicating a moderate to high degree of overall accuracy.

The DOR is an overall measure of test accuracy that combines data from sensitivity and specificity into a single indicator. It is calculated as the ratio of the odds of positivity results in the diseased persons relative to the odds of positivity results in the nondiseased individuals.^32^ The value of DOR ranges from 0 to infinity. A test with a higher DOR value indicates a better discriminability. A DOR of 1.0 indicates that a test has no discriminability between patients with the disease and individuals without it.^32^ In our study, we report a mean DOR of 22.55, confirming a moderate to high level of overall accuracy.

Although the conclusions drawn from pooling likelihood ratios are similar to those obtained by pooling sensitivities and specificities, the likelihood ratios can be more easily interpreted compared with the SROC curve and the DOR and more meaningful to clinical practice. Both PLR and NLR were used as our measures of diagnostic accuracy. A PLR value of 4.94 means that patients with SBP have an ∼ 5-fold higher chance of being PCT test-positive compared with those without SBP. By comparison, the NLR was found to be 0.22 in our meta-analysis, meaning that if the PCT test result was negative, the probability that this patient suffers SBP is ∼20%. So, on the basis of the currently available data, both positive and negative likelihood ratios were not high or low enough to be used as a valuable tool for rule-in and rule-out SBP diagnosis. The results of the assay must therefore be interpreted prudently in combination with the microbiological finding, medical history, and physical examination.

Consistent with the previous report,^12^ we observed a substantial degree of heterogeneity. Our meta-analysis should be interpreted in light of several limitations. First, the different etiologies of liver diseases and the varying severity of liver function in different studies were a source of heterogeneity. Theoretically, this potential heterogeneity can be explored with individual patient data or analyzed by meta-regression analysis. However, the absence of these data and the limited number of articles that met our eligibility criteria makes our endeavor to explore the sources of heterogeneity impossible. Second, included studies in our meta-analysis covered a wide range of cut-off values, which may lead to sensitivities and specificities vary greatly across studies and negatively correlate with each other due to the threshold effect. Third, the limited number of studies and small pooled sample size may also impose a limitation on our study. The limited number of articles included in our meta-analysis prevented us from performing more specific subgroup, and small sample sizes of included studies may be easily lead to type II error and wide CIs. Also, we could not evaluate the influence of aspects such as expertise with PCT assay technology, laboratory infrastructure, and setting on the accuracy of the PCT measurements due to the lack of required records in the original papers. Another limitation of our analysis was the poor quality of the evidence. There was no randomized control trial and even 3 retrospective studies included. Strict methodological criteria means that only minority of available studies can be included. Alternatively inclusion of many imperfect studies may have led to an overestimation of the diagnostic accuracy. A definitive conclusion was difficult to make from the less precise pooled estimates.

In conclusion, early identification of SBP due to ESLD remains a challenge for clinicians. A marker specific for bacterial infection would be desirable. Based on the results of the present meta-analysis, serum PCT may be used as an aid for clinicians to rapidly and accurately diagnose SBP. An algorithm that integrates the clinical information and PCT results may provide a useful indicator of when to initiate and continue antibiotic therapy. Given the small number of publications on this subject, more large, prospective, well-designed studies are needed to access the diagnostic accuracy of PCT in SBP due to ESLD.
